# The Ophthalmoscopic Appearances in Cases of Exophthalmic Goitre

**Published:** 1873-08

**Authors:** Reuben A. Vance

**Affiliations:** New York City


### Chicago Medical Journal.

A MONTHLY RECORD OF

*Medicine, Surgery and the Collateral Sciences.*

EDITED BY J. ADAMS ALLEN, M.D., LL.D.; AND WALTER HAY, M.D.

---

VOL. XXX.—AUGUST, 1873.—NO. 8.

---

### Original Communications.

ARTICLE I.—*The Ophthalmoscopic Appearances in Cases of Exophthalmic Goitre.* By REUBEN A. VANCE, M.D., New York City.

This peculiar neurosis, like many other affections of the nervous system, is attended with morbid changes in the intra-ocular structures which can be recognized by the aid of the ophthalmoscope. The symptoms of the disease, however, are so marked, the exophthalmos and enlargement of the thyroid gland, with palpitation of the heart, so characteristic, that the diagnosis of the affection requires no aid from this method of physical exploration. Yet, so long as there are unsettled points, or diverse views, in relation to the pathology of any morbid process, attention to the visible phenomena and careful observation of those changes which can be rendered apparent, should not be neglected. This is especially the case where nervous structures are involved, either primarily or secondarily, and is my reason for calling attention to the ophthalmoscopic appearances presented in ten cases of this disease which have been under my care at various times during the past five years. The intra-ocular structures were affected as follows:

In 2 cases there was anæmia;

In 7 cases there was congestion; and

In 1 case there was congestion, with œdema of the disk.

The patients were all under thirty years of age; eight were females and two were males; organic disease of the heart was discovered in six cases—one male and five females; the three characteristic symptoms, exophthalmos, enlargement of the thyroid gland, and palpitation, were noted in all but one case—in this patient the pulse was 120 when in repose, the thyroid was quite large and palpitation of the heart so distressing as to seriously interfere with sleep, yet the eyes did not protrude ordinarily, but only became prominent under excitement.

The intra-ocular morbid appearances are mainly those due to vascular disturbance of the parts in the region of the optic nerve entrance. In nine cases they were limited to variations in the calibre, course, and state of distension of the blood-vessels of the papilla and retina, while in one, these changes were supplemented by an œdematous state of the disk in the right eye, the left remaining congested. The ophthalmoscopic appearances were modified quite appreciably, in cases of congestion, by mental excitement, or physical exertion, but in anæmic cases, such influences produced no perceptible changes, although the exophthalmos was invariably increased, and the other phenomena of the disease were rendered more apparent.

In two cases the state of vascular fullness was much less than in health. The retinal arteries were small and indistinct, straight in their course and joined by few branches; the veins were likewise diminished in size, were less darkly colored than in health, and presented the double outline generally observed in the arteries only; the disk was not of its natural color, was unusually transparent, and with the direct examination, its fibrous structure could be readily discerned, while the vascular branches apparent in health had disappeared from view. In these cases physical examination could detect no cardiac disease of an organic nature, and, as has already been stated, neither mental excitement nor physical exertion had any effect upon the character of the intra-ocular appearances.

In the cases of congestion, the ophthalmoscope revealed appearances directly the reverse of those just described. The vascular supply of the intra-ocular structures was in excess of the standard of health, and varied in degree from slight hyperæmia to congestion so extensive as to render the papilla of the same color as the surrounding choroid and retina. The arteries distributed to the retina were larger than in health, their branches more numerous and their course less direct; the disk was variously colored, at times its normal creamy-white hue was changed to a rosy tint, and again it assumed the same color as the surrounding structures—in such cases its situation could be discovered only by tracing the retinal vessels to their point of exit; the veins were dilated and tortuous, and at the points where they were joined by branches, the main trunks presented irregular enlargements, in some cases closely simulating hæmorrhagic extravasations. New vessels were developed from the sides of the disk, and subdivided into branches of considerable size. Venous pulsation, especially in the main vessels as they traversed the papilla, was commonly observed, and in one patient, well marked arterial dilatation and contraction synchronous with the cardiac systole and diastole, was a constant phenomenon.

In one patient, presenting marked evidences of intra-ocular hyperæmia, an examination made a few hours subsequent to a violent paroxysm of dyspnœa, attended with spasm of the glottis, violent palpitation and great mental excitement, revealed congestion with œdema of the disk in the right eye, while the congestive appearances in the left were unchanged. The right disk, which previously had been of a deep red color, was opaque and striated, grayish-white in appearance and irregularly enlarged—its nasal side projecting abruptly, the infiltration gradually diminishing towards the temporal margin where it was in the same plane as the adjacent retina. The retinal veins were quite large, tortuous, and presented circumscribed dilatations, while the arteries were nearly normal in appearance—much smaller than they had been previously, or than those of the left eye.

The generally accepted view of the pathology of this disease as a congestive neurosis, attended with secondary changes in the tissues of the organs principally affected—the walls and orifices of the heart, the substance of the thyroid gland, and the intra-orbital structures—would account for the ophthalmoscopic appearances in the majority of the cases I have referred to. The increased throbbing of the thyroid, the greater projection of the eyes, and the distressing palpitation under emotional or physical excitement, coincide so fully with the results of experiments performed on the sympathetic, together with its known physiological action in parts where its effects can be observed, renders the inference almost irresistible, that to perverted activity of that nerve, is to be ascribed the curious phenomena of exophthalmic goitre. The congestive changes due to vaso-motor paresis would explain the post-mortem appearances observed in the thyroid, the heart, and the parts behind the eye, and, as one would imagine, the changes rendered visible, during life, by the aid of the ophthalmoscope. That it does not do so in all cases, the patients presenting evidences of intra-ocular anæmia that I have alluded to, show most conclusively. It is possible that some local change, by interfering with the intra-ocular circulation, may produce this anæmic state, and at one time, from certain other circumstances, I was led to believe that secondary changes in the congested tissues behind the eye, compressing the nervo-vascular trunk, was the cause of the intra-ocular appearances in these exceptional cases. Notwithstanding the many objections that can be readily urged against this explanation—the most cogent of which is the peculiar nature of the ophthalmoscopic appearances themselves—I am disposed to adhere to it until some better one is offered.

In the remaining cases the intra-ocular evidences of congestion in its various degrees, were observed to fluctuate with exacerbations and remissions of the disease. An increase in the severity of the symptoms was accompanied by an increased degree of intra-ocular hyperæmia, and *vice versa.* In one instance œdema of the disk was observed as a consequence of an extreme degree of congestion, and this circumstance is of importance as an illustration of the fact that that condition can be developed from local derangement of the circulation, and is not invariably of intra-cranial origin as is frequently stated.

